# Catquest-9SF questionnaire and eCAPS: Validation in a Canadian population

**DOI:** 10.1371/journal.pone.0237788

**Published:** 2020-09-25

**Authors:** Matthew B. Schlenker, Simona C. Minotti, Anna Kabanovski, Morgan Lim, Chelsea D’Silva, Julia Ma, Robert Reid, Iqbal Ike K. Ahmed

**Affiliations:** 1 Institute for Better Health, Mississauga, Ontario, Canada; 2 Department of Ophthalmology and Vision Sciences, University of Toronto, Toronto, Ontario, Canada; 3 Department of Statistics and Quantitative Methods, University of Milano-Bicocca, Millan, Italy; 4 Faculty of Medicine, University of Toronto, Toronto, Ontario, Canada; 5 Institute of Health Policy, Management and Evaluation, University of Toronto, Toronto, Ontario, Canada; 6 Trillium Health Partners, Mississauga, Ontario, Canada; University of Copenhagen, DENMARK

## Abstract

**Background:**

Visual acuity alone has limitations in assessing a patient’s appropriateness and prioritization for cataract surgery. Several tools, including the Catquest-9SF questionnaire and the electronic cataract appropriateness and priority system (eCAPS) have been developed to evaluate patients–reported visual function as related to day-to-day tasks. The aim of this study was to validate Catquest-9SF and eCAPS in a Canadian population and propose a shorter version of each, in an attempt to extend their applicability in clinical practice.

**Methods:**

The English translation of the Swedish Catquest-9SF and eCAPS were self-administered separately in pre-operative patients in tertiary care in Peel region, Ontario. Rasch analysis was used to validate both scales and assess their psychometric properties, such as category threshold order, item fit, unidimensionality, precision, targeting, and differential item functioning.

**Results:**

A total of 313 cataract patients (mean age = 69.1, 56.5% female) completed the Catquest-9SF and eCAPS. Catquest-9SF had ordered response thresholds, adequate precision (person separation index = 2.09, person reliability = 0.81), unidimensionality and no misfits (infit range 0.75–1.35, outfit range 0.83–1.36). There mean for patients was equal to -1.43 (lower than the mean for items which is set automatically at zero), meaning that tasks were relatively easy for respondent ability. eCAPS had 3 items that misfit the Rasch model and were excluded (infit range 0.82–1.30, outfit range 0.75–1.36). Precision was inadequate (person separation index = 0.19, person reliability = 0.04). 78.8% of subjects scored≤9 (answered that they had no issues for most questions).

**Conclusions:**

Catquest-9SF demonstrated good psychometric properties and is suitable for assessing visual function of care-seeking patients referred for cataract surgery in Ontario, Canada. There was some mistargeting, suggesting that the tasks were relatively easy to perform, which is consistent with previous research. On the contrary, eCAPS is not sensitive in differentiating patients who had impaired visual functioning.

## 1. Introduction

Cataracts are the most common cause of treatable blindness [1]. Currently, cataract surgery is the only effective treatment and is one of the most frequently performed surgical procedures in Canada and worldwide [2]. As people live longer, the prevalence of cataracts is growing and the demand for cataract surgery is on the rise. For instance, in Ontario alone, the number of cataract surgeries is projected to increase more than 2-fold from 143,000 in 2006–2007 to 326,000 by 2036 [2].

With increasing demand of cataract surgery, there is a growing need to accurately evaluate appropriateness for cataract surgery, determine prioritization on waiting lists, and quantify surgical outcomes. Traditionally, ophthalmologists have used high contrast distance visual acuity and other objective clinical measures to assess appropriateness, prioritization, and outcomes. Although these “hard measurements” are critical, other factors are important to patients as well, such as brightness, contrast, colour discrimination, glare, and ultimately visual function as related to daily tasks [3–5]. Thus, health-related quality of life (HRQOL) survey instruments have been developed to evaluate patient reported visual function. Examples of vision-specific instruments include the Catquest, Activities of Daily Vision Scale (ADVS), Visual Functioning 14 (VF-14), Glaucoma Utiliy Index, NEI-visual functioning questionnaire 25 (NEI-VFQ), and Cat-PROM5, among many others [6–11].

Most of these self-rated questionnaires were developed in a traditional psychometric paradigm framework called Classical Test Theory (CTT) [12, 13]. More recently, Item Response Theory (IRT) has become a widely used alternative methodology to CTT. The methodology of choice relies on the analysts’ purpose and preferences and is a matter of debate [14]. Although CTT holds fewer assumptions and is less computationally complex, IRT may have some advantages in certain circumstances, particularly where there is less confidence about the items, and there is a need to address dimensionality, differential item functioning, and item fit [14]. Spurious relationships can also arise during stepwise modelling, that then must be addressed [15]. One limitation of CTT is that it uses summary scoring—simple addition of values assigned to responses [10, 12]. Since steps along the continuum often have unequal sizes, summary scoring may not be an ideal approach. IRT overcomes this limitation by converting ordinally arranged data to an interval scale where steps are the same size. This accounts for items having varying difficulties and allows addition of scores and use of parametric statistical analysis. Another reason to utilize the IRT model is that respondents may complete different sets of items without affecting score accuracy, whereas with CTT, an easier set of items will yield an overestimated score of person ability [16]. This feature of IRT led to the development of computerized adaptive testing (CAT), a system which tailors items for each respondent based on prior answers and person ability. CAT aims to improve precision, targeting, and efficiency, and an area of future research for our group. Within IRT, the Rasch model has been widely used in patient-reported outcome questionnaire development due to its relative simplicity compared to other IRT models [16, 17]. For these reasons, Rasch modeling is particularly useful in the development and validation of patient-reported outcome questionnaires.

An example of a questionnaire validated and optimized through Rasch Analysis is the Catquest questionnaire, which is a commonly used tool that evaluates vision-related limitations in performing day-to-day tasks. Originally developed in 1995 using a CTT framework based on data from the Swedish National Cataract Register [17], in 2009 it was revised through Rasch analysis to create a new 9-item version called the Catquest-9SF (short-form) questionnaire [18]. The survey is short, easy to use, and cost-effective to administer, making it a practical tool to implement in daily clinical practice. First validated in Sweden, the Catquest-9SF has been translated, culturally adapted, and validated in Australia, Austria, China, Denmark, England, Germany, Italy, Malaysia, the Netherlands, and Spain [11, 18–30]. The Catquest-9SF has been shown to be a reliable instrument for measuring visual function and is highly responsive to cataract surgery outcomes in multiple languages [30]. However, it has not yet been validated in Canada.

In Alberta, Canada, the Western Canada Wait List Project (WCWLP) developed a measurement instrument to assess cataract surgery priority criteria [31]. This tool was recently modified in Ontario, Canada to create another measurement instrument for patient reported visual function—the electronic Cataract Appropriateness and Prioritization System (eCAPS) [32]. eCAPS aims to assess appropriateness for cataract surgery and prioritize patients for the surgery based on clinical and patient-rated HRQOL criteria (S2 Fig). A modified Delphi process, which is a structured method of group communication with online anonymous surveys, was conducted with nine ophthalmologists, three optometrists, and one family physician to choose the criteria to be included in the eCAPS tool, resulting in a 10-item questionnaire (S2 Fig). eCAPS was found to have good inter- and intra-rater reliability but has not yet been validated for clinical use.

In this study we assess whether the Catquest-9SF and eCAPS questionnaires are valid and suitable for use in clinical practice in pre-operative patients with cataract in Peel region, Ontario, Canada. In addition, we propose a shorter version of the two questionnaires, in an attempt to extend their applicability in clinical practice.

## 2. Methods

### 2.1. Catquest-9SF questionnaire

The Catquest-9SF is a 9-item Rasch-scaled questionnaire [18]. It comprises 2 global assessment questions (Ca and Cb) and 7 questions related to specific daily-life activities (C1-C7). Each item has four response options. For questions Ca and C1-C7, the options are: 4 = ‘Yes, very great difficulties’; 3 = ‘Yes, great difficulties’; 2 = ‘Yes, some difficulties’; and 1 = ‘No, no difficulties’. For question Cb, which asks about satisfaction with vision, the response options are: 4 = ‘Very dissatisfied’; 3 = ‘Rather dissatisfied’; 2 = ‘Fairly satisfied’; 1 = ‘Very satisfied’. All items also contain a ‘Cannot decide’ option. The English translation of the Swedish nine-item Catquest-9SF was used. This version was appropriate for use in Canada, so there were no adaptations apart from slightly different wording of questions to improve clarity (S1 Fig). The questionnaire is publicly available through the International Consortium for Health Outcomes Measurement (ICHOM) standard set for cataracts. A license is not required for use.

### 2.2. eCAPS

eCAPS has two components—a clinical questionnaire and quality of life questionnaire. The clinical questionnaire was excluded from this analysis; only the quality of life patient reported questionnaire was used. It has 10 items with three ordinal response options for each item (S2 Fig). The first two questions (E1, E2) ask to rate the extent of impairment in visual function and other substantial disabilities. The response options are: ‘none’; ‘mild/moderate’; or ‘severe’. The other 8 questions (E3-E10) focus on abilities in daily-living tasks. The response options are: ‘not threatened’; ‘mildly/moderately threatened’; or ‘severely threatened’.

### 2.3. Participants

Participants were recruited through 3 clinics at Prism Eye Institute and one private ophthalmologist’s office in Peel region, Ontario, Canada between September 2016 and May 2017. Patients who were referred for cataract surgery who were English-speaking and were aged 40–85 were eligible to participate in the study. Patients with cognitive impairment as measured by the Short Orientation Memory Concentration Test, patients with a multifocal lens, or patients with a prior cataract surgery within the last 4 months were excluded [33]. This study was approved by the Trillium Health Partners Research and Ethics Board and written consent was obtained from each participant. Participants completed the Catquest-9SF and eCAPS questionnaires separately before surgery. Both questionnaires were self-administered.

### 2.4. Rasch analysis

IRT methodology, specifically Rasch analysis, was the chosen approach for validation of Catquest-9SF. In addition to considering the advantages of the approach as described above, we chose this route because Catquest-9SF was developed and previously validated in 11 countries using the Rasch analysis [30]. Therefore, using the same technique will allow us to ensure comparability with previous studies and maintain standardized methods of analysis for this questionnaire, and position us to consider CAT approaches in the future. Given this decision, we opted to use the Rasch analysis for eCAPS as well which will allow an accurate analysis and consistency of methodology throughout the paper.

Rasch analysis was performed on data obtained from Catquest-9SF and eCAPS tools separately. Rasch analysis places a person’s ability to perform a task (in this case, their visual function) with the level of difficulty required to perform that task (item difficulty) on the same linear scale, measured in log of the odds (logit) units [34]. A person with a higher ability and an item with a greater difficulty is expressed on the negative side of the logit scale. For example, in a representation of the logit scale such as a person-item map, a person placed at the most negative part of the scale has high visual function and likely responded ‘no, no difficulties’ or ‘not threatened’ to all questions. A person placed at 0 responded that they had some difficulties performing some of the tasks, likely the more difficult ones placed at the more negative part of the scale. Therefore, the Rasch model gives information regarding how well items fit the trait being measured (in this analysis, visual function) and the scale’s ability to distinguish respondents based on ability. Rasch analysis was performed with Winsteps software (v.4.4.4) [35]. Psychometric properties were assessed with category threshold order, misfitting items, unidimensionality, precision, targeting, and differential item functioning (S1 Table) [36–43]. The option “cannot decide” on Catquest-9SF is treated as missing in the standard Rasch analysis, meaning that those answers are simply not included in the likelihood function [19, 24, 28, 29]. This approach to handling the missing data is accurate because the Rasch model can account for it and imputation is not required if the data fit the model [44, 45]. As a sensitivity analysis, we excluded respondents who chose ‘cannot decide’ for any of the items on Catquest-9SF and re-ran the Rasch analysis.

### 2.5. Validation of a subset of Catquest-9SF questions

Previous validation studies of Catquest-9SF demonstrated clustering of items on the person-item map, suggesting that some questions may be redundant [18–20, 22, 23, 27–29]. In this study, once the above analysis was performed for the 9-item Catquest questionnaire, we noticed that there were two clusters of three items on the person-item map. This finding, in addition to the practical considerations of the length of the survey, encouraged us to attempt to shorten the Catquest questionnaire by removing some of the potentially redundant items. We removed two items from each cluster (yielding a 5-item questionnaire) and re-ran the Rasch analysis. We also checked combinations with one item removed from each cluster (yielding a 7-item questionnaire) and one item removed from either cluster (yielding an 8-item questionnaire).

## 3. Results

### 3.1. Participants

A total of 539 patients were approached to participate in the study. Of those that were approached, 313 patients (58.1%) consented and returned the Catquest-9SF and eCAPS questionnaires. The median age of participants was 70.0 (mean = 69.0, SD = 8.29) and 56.5% were female (Table 1). 47.2% had pre-operative vision of 20/50 and better.

**Table 1:** Participant demographics.

**Age (n = 312)**	
Average	69.0
Range	42–84
**Gender (n = 313)**	
Female	177 (56.5%)
Male	136 (43.5%)
**Ethnicity (n = 312)**	
Africa	12 (3.9%)
Americas	85 (27.4%)
Asia	88 (28.4%)
Europe	125 (40.3%)
**Household Income (n = 189)**	
<$30,000	45 (23.8%)
$30,000-$49,999	29 (24.4%)
$50,000-$69,999	30 (15.9%)
$70,000+	66 (34.9%)
**Education (n = 310)**	
Less than high school	48 (15.4%)
High school	84 (26.9%)
Apprenticeship	16 (5.1%)
College	66 (21.1%)
University	98 (31.4%)
**Pre-operative best corrected visual acuity—eye for surgery (n = 312)**	
20/30 or better	63 (20.2%)
20/40-20/50	85 (27.2%)
20/60-20/150	103 (33.0%)
20/200 or worse	61 (19.6%)

A total of 313 patients were included (n < 313 means data was not reported by patients).

### 3.2. Catquest-9SF

Overall Catquest-9SF met criteria for acceptable category threshold order, fit statistics, and precision. Unidimensionality was confirmed and there was lack of notable differential item functioning. There was some mistargeting, indicating that the items were relatively easy for respondent ability. The results for all criteria are outlined below.

#### 3.2.1. Threshold order

**Fig 1:**
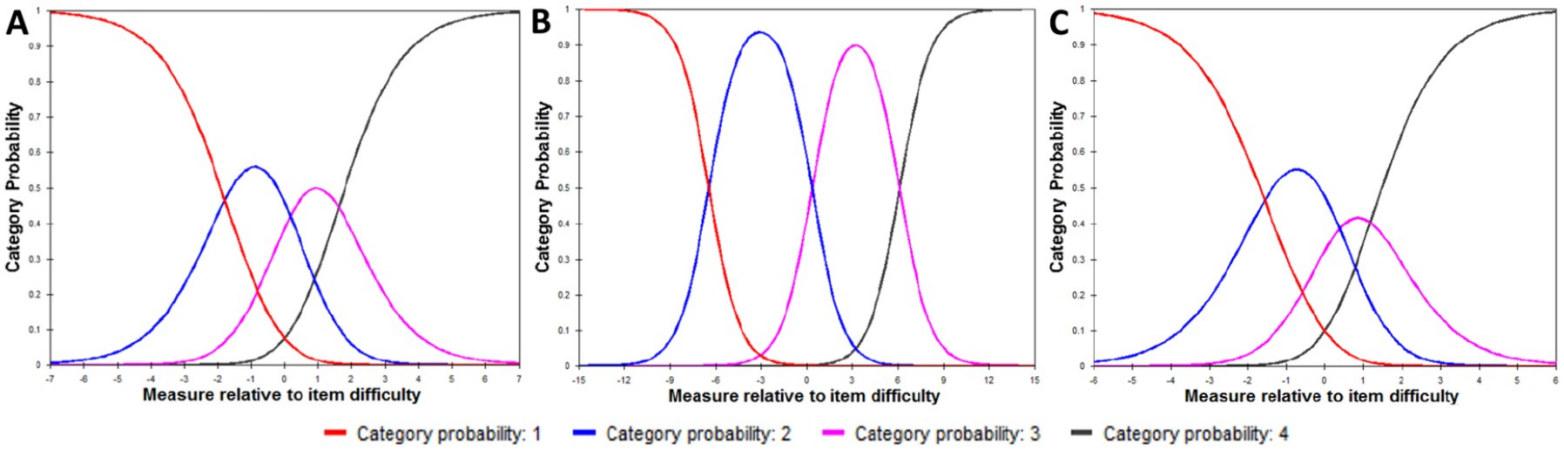
Category probability curves for Catquest-9SF. A) all 9-items, B) global assessment items Ca and Cb only, and C) daily-life activities items C1-C7 only.

As shown in the category probability curves (Fig 1), the thresholds were ordered for all questions.

#### 3.2.2. Item calibration and fit

All items fit into the Rasch model, with infit range 0.75–1.35 and outfit range 0.83–1.36 (Table 2). This is within the acceptable range of 0.50–1.50.

**Table 2:** Rasch analysis results of full Catquest-9SF.

Item	Question	Item Calibration† (SE)	Infit MNSQ	Outfit MNSQ
Ca	Difficulties in daily life	-0.09 (0.09)	0.75	0.89
Cb	Satisfaction with vision	-2.27 (0.09)	0.91	1.09
C1	Read newspaper text	-0.67 (0.09)	1.00	0.95
C2	Recognize faces	1.45 (0.11)	1.27	1.01
C3	See prices when shopping	-0.41 (0.09)	0.91	0.85
C4	Walk on uneven ground	0.79 (0.10)	1.35	1.36
C5	Do needlework/handicraft	0.69 (0.10)	1.23	1.04
C6	Read text on television	-0.25 (0.09)	0.89	0.83
C7	Carry out a hobby	0.76 (0.10)	1.08	0.85

^†^Measured in logits. A positive value indicates that the item is easier (requires lower visual function) while a negative value indicates that the item is more difficult (higher visual function is required).

#### 3.2.3. Unidimensionality

Principal component analysis of the residuals for the Catquest-9SF showed that the variance explained by the measures was comparable for the empirical calculation (58.3%) and by the model (59.0%). The observed unexplained variance was 41.7%, which is comparable to the unexplained variance expected if the data fit the Rasch model perfectly (41.0%). The unexplained variance explained by the first contrast was 1.7 eigenvalue units, which is lower than 2.0 and is therefore close to the magnitude seen with random data.

#### 3.2.4. Precision

Person separation index and person reliability were 2.09 and 0.81, respectively, indicating that the instrument had acceptable capability to discriminate respondents based on their abilities (minimum acceptable values are 2.00 and 0.80, respectively, for differentiating between low, medium, and high ability) [34]. Cronbach’s alpha was 0.87, indicating that the questionnaire has good internal consistency (0.70 to <0.80 is acceptable, 0.80 to <0.90 is good, and greater than 0.90 is excellent) [11, 29].

#### 3.2.5. Targeting

The mean person location was -1.43, which is substantially lower than the mean for items (set automatically to be zero). This indicates some mistargeting, meaning that the items were relatively easy for respondent ability. That is, respondents were more likely to indicate that they had no or some difficulties with the tasks on the questionnaire.

#### 3.2.6. Differential item functioning (DIF)

There was no significant DIF for Catquest-9SF for age or gender. Minimal DIF (defined as DIF contrast between 0.50 and 1.0, with p<0.05) occurred as a function of pre-operative visual acuity for the item C5 (‘do needlework/handiwork’) (DIF contrast = 0.56, p = 0.0069, rated more difficult by those with worse pre-operative vision).

#### 3.2.7. Person-item map

The easiest question was C2 (‘recognize faces’) only respondents with very low visual function are unable to recognize faces. The most difficult question was Cb (‘satisfaction with vision’), meaning that visual function does not have to be very low for people to state that they are not satisfied with their vision.

**Fig 2:**
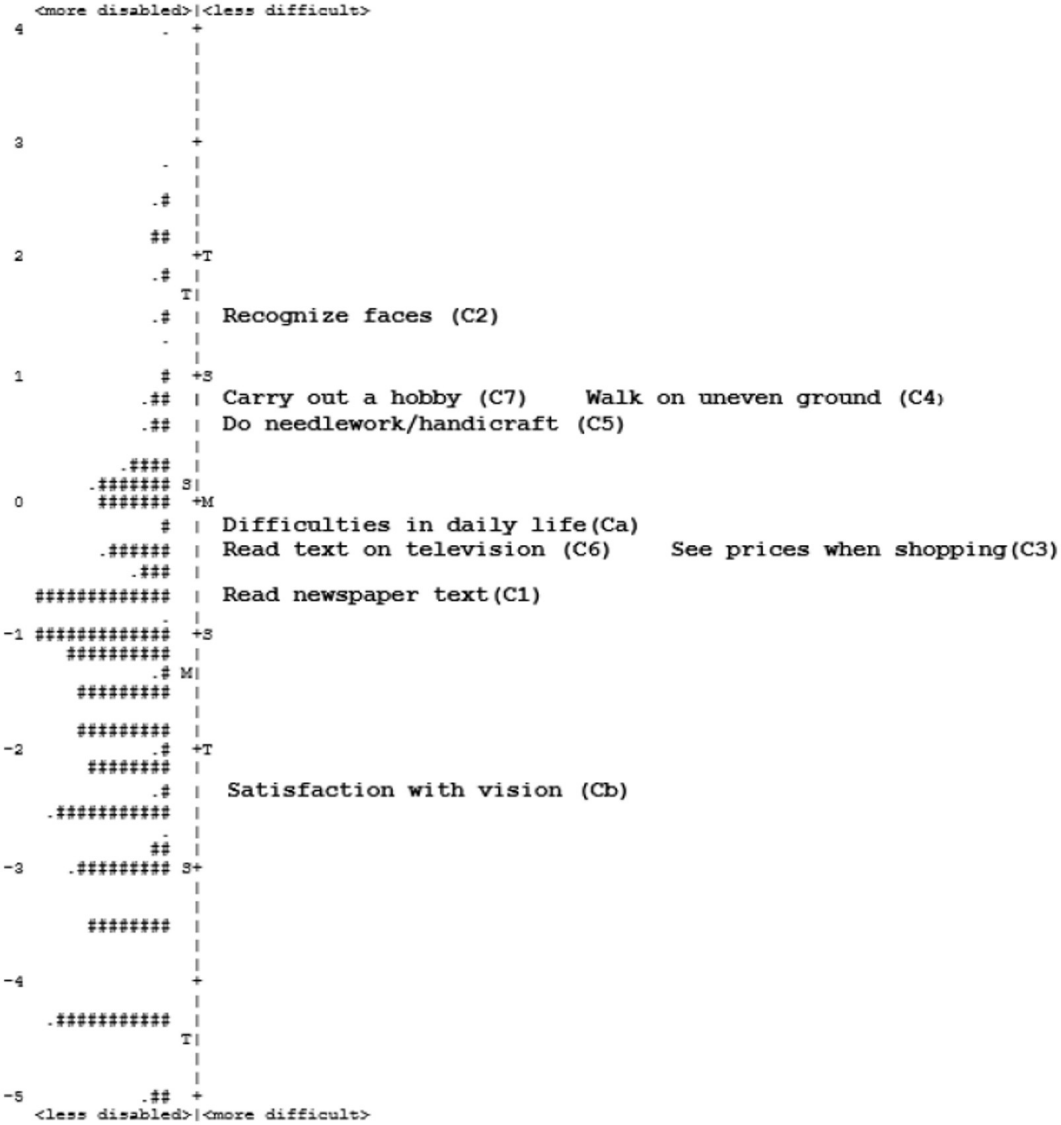
Person-item map for Catquest-9SF. Respondents are shown on the left side of the dashed line and items are shown on the right side. Respondents who are less disabled (have higher visual function) and items that are more difficult (respondents are more likely to rate having greater difficulties with tasks) are located at the bottom. Each ‘#’ represents 2 respondents and each ‘.’ represents 1 respondent. M = mean, S = 1 standard deviation, T = 2 standard deviations. The scale is in logits.

There were two clusters of three items, as shown on the person-item map (Fig 2). The first cluster includes items C4 (‘walk on uneven ground’), C5 (‘do needlework/handicraft’), and C7 (‘carry out a hobby’). The second cluster includes items Ca (‘difficulties in daily life’), C3 (‘see prices when shopping’) and C6 (‘read text on television’). Items in the same cluster likely measure a similar level of visual function. For example, needlework/handicraft may fall under hobbies for some respondents, and many people may say that trying to see prices is like trying to see text on television.

#### 3.2.8. Sensitivity analysis

We performed a Rasch analysis on the Catquest-9SF data excluding respondents who answered ‘cannot decide’ for any of the items. 274 participants were included for this analysis and the results were largely the same. Infit range was 0.74–1.36, outfit range 0.83–1.40 with no misfitting items. Person separation index was 2.17 and person reliability was 0.83. The mean person location was 1.43 logits below the mean item location and there were no significant differences on the person-item map. There were no instances of differential item functioning with respect to age or gender.

#### 3.2.9. Subsets of questions

As discussed, two clusters of items are evident on the person-item map of Catquest-9SF: items C4, C5, and C7 and items Ca, C3 and C6. Psychometric properties of 5, 7, and 8-item combinations are shown in S2 Table.

Psychometric properties were acceptable for all shortened versions. Category thresholds were ordered, the infit and outfit ranges were within the acceptable range (0.50–1.50) with no misfitting items, and unidimensionality was confirmed by principal components analysis. All combinations showed mistargeting with a difference between the mean for persons and mean for items >1.0, indicating the items were too easy to perform. There was no DIF of magnitude >0.50 with age or gender.

Reducing the number of questions adversely affected precision. The 8-item combinations with items C4, C5, or C7 removed had acceptable precision, with the highest precision in the combination excluding item C4 (included Ca, Cb, C1, C2, C3, C5, C6, C7). However, precision was unacceptable for all 5- and 7-item shortened versions of the questionnaire that were tested, as in person separation index and person reliability values were lower than 2.00 and 0.80, respectively. The 7-item combination that had the highest precision was items Cb, C1, C2, C3, C5, C6, and C7 (items Ca and C4 removed). The 5-item combination with the highest precision was items Cb, C1, C2, C3, C7 (items Ca, C4, C5, C6 removed). The combination with the highest precision among the 5 and 7-item questionnaires, as well as the 8-item combinations with acceptable precision are shown in Table 3.

**Table 3:** Psychometric properties of combinations of Catquest-9SF with best Rasch results.

Number of Items Remaining	5	7	8	8	8
**Removed Items**	Ca, C4, C5, C6	Ca, C4	C4	C5	C7
**Infit Range**	0.86–1.35	0.90–1.31	0.79–1.35	0.72–1.40	0.75–1.33
**Outfit Range**	0.80–1.23	0.82–1.37	0.86–1.18	0.83–1.38	0.84–1.31
**Variance explained by the measures for empirical calculation; for model (%)**	66.8; 67.0	61.6; 62.4	60.4; 61.1	59.7; 60.3	59.2; 59.5
**Unexplained variance explained by the first contrast (eigenvalue units)**	1.61	1.63	1.67	1.65	1.69
**Person Separation Index (PSI)**	1.77	1.97	2.09	2.03	2.00
**Person Reliability (PR)**	0.76	0.80	0.81	0.81	0.80
**Difference between mean for persons and mean for items**	-1.37	-1.39	-1.40	-1.39	-1.36

Combinations have 5 items remaining (4 items removed—two from each cluster), 7 items remaining (2 items removed—one from each cluster), and 8 items remaining (1 item removed—from either the first or second cluster).

### 3.3. eCAPS

Overall, three items were excluded due to unacceptable fit statistics. The remaining 7-items did not have adequate precision, meaning that the instrument cannot separate respondents based on ability. Rasch-based metrics are reported below.

#### 3.3.1. Threshold order

**Fig 3:**
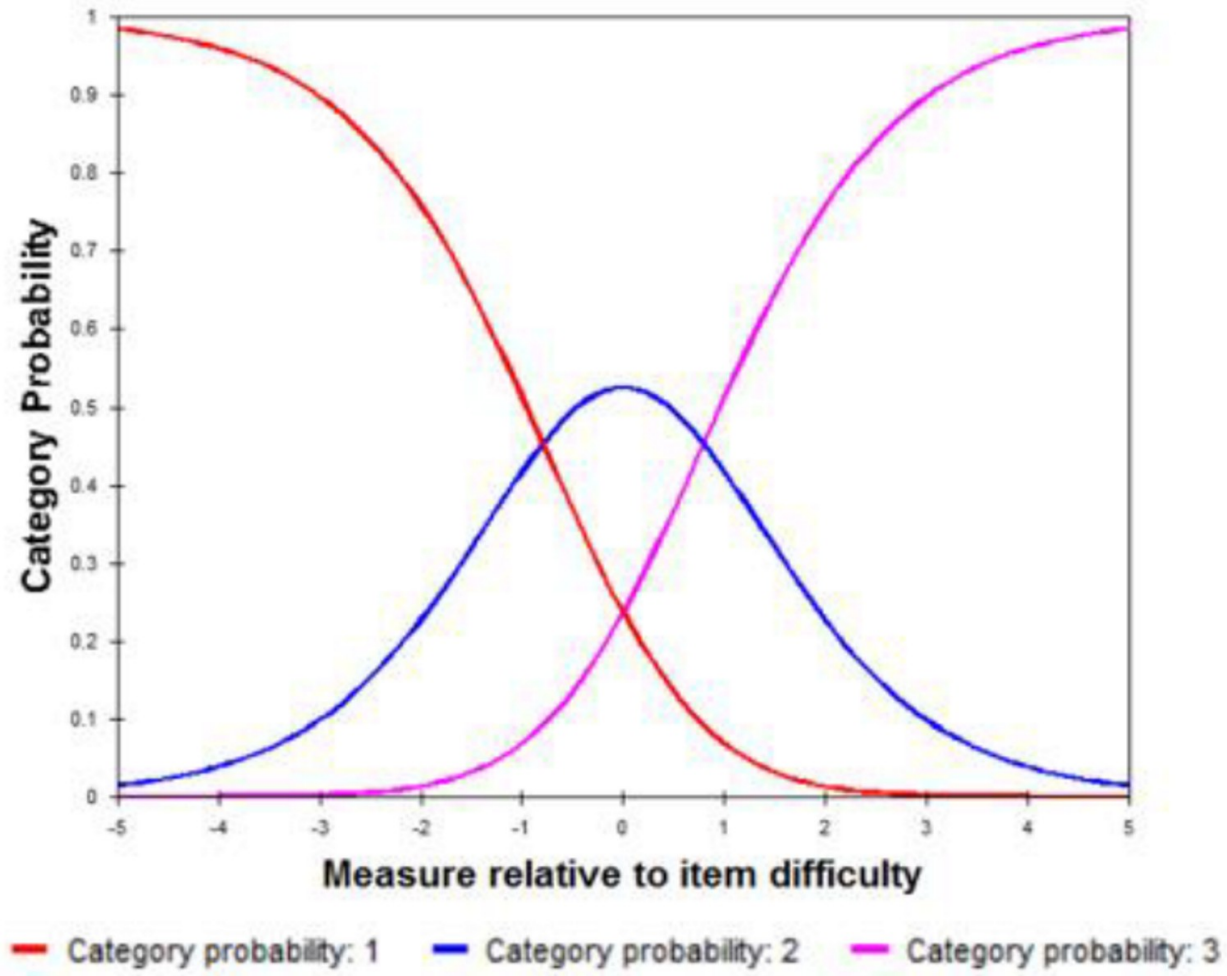
Category probability curve for eCAPS questionnaire.

As shown in the category probability curve (Fig 3), the response thresholds were ordered.

#### 3.3.2. Item calibration and fit

Three items misfit the model and were excluded from the remainder of the analysis: E1-‘extent of impairment in visual function’, E2-‘other substantial disabilities’, and E7-‘ability to take care of your own health’, which had outfit MNSQ values of 1.76, 1.72, and 0.43, respectively. The remainder of the items fit well into the model with infit range 0.82–1.30 and outfit range 0.75–1.36, which is within the acceptable 0.50–1.50 range (Table 4).

**Table 4:** Rasch analysis results of eCAPS questionnaire.

Item	Question	Location (SE)†	Infit MNSQ	Outfit MNSQ
**E1**	Extent of impairment in visual function	Removed		
**E2**	Other substantial disabilities	Removed		
**E3**	Safety and injury concerns	-0.83 (0.13)	1.30	1.36
**E4**	Ability to work, care for dependents	-0.13 (0.15)	1.00	0.96
**E5**	Ability to take care of local errands	-0.31 (0.15)	0.89	0.87
**E6**	Ability to take care of household business	1.01 (0.20)	0.82	0.85
**E7**	Ability to take care of own health	Removed		
**E8**	Ability to provide assistance to others	0.60 (0.18)	0.89	0.75
**E9**	Ability to participate in social life	0.24 (0.17)	0.85	0.75
**E10**	Take part in active recreational activity	-0.59 (0.14)	1.13	1.11

^†^Measured in logits. A positive value indicates that the item is easier (requires lower visual function) while a negative value indicates that the item is more difficult (higher visual function is required).

#### 3.3.3. Unidimensionality

Principal component analysis of the residuals for the eCAPS showed that the variance explained by the measures was comparable for the empirical calculation (33.9%) and by the model (35.3%). The observed unexplained variance was 66.1%, which is comparable to the unexplained variance expected if the data fit the Rasch model perfectly (64.7%). The unexplained variance explained by the first contrast was 1.48 eigenvalue units, which is less than 2.0 (noise level). These values confirm unidimensionality of eCAPS and that there was no evidence of another latent trait captured by the scale. Cronbach’s alpha was 0.78, indicating that the questionnaire has acceptable internal consistency [11, 29].

#### 3.3.4. Precision

Person separation index and person reliability for eCAPS were 0.19 and 0.04, respectively. These values are too low, indicating that the instrument utilized in this population is unable to separate respondents based on their abilities. 78.8% of subjects scored 9 or below, meaning they responded with ‘none/not threatened’ for almost all questions. The question that demonstrated the best spread in responses was E1—‘extent of impairment in visual function.’

#### 3.3.5. Targeting

**Fig 4:**
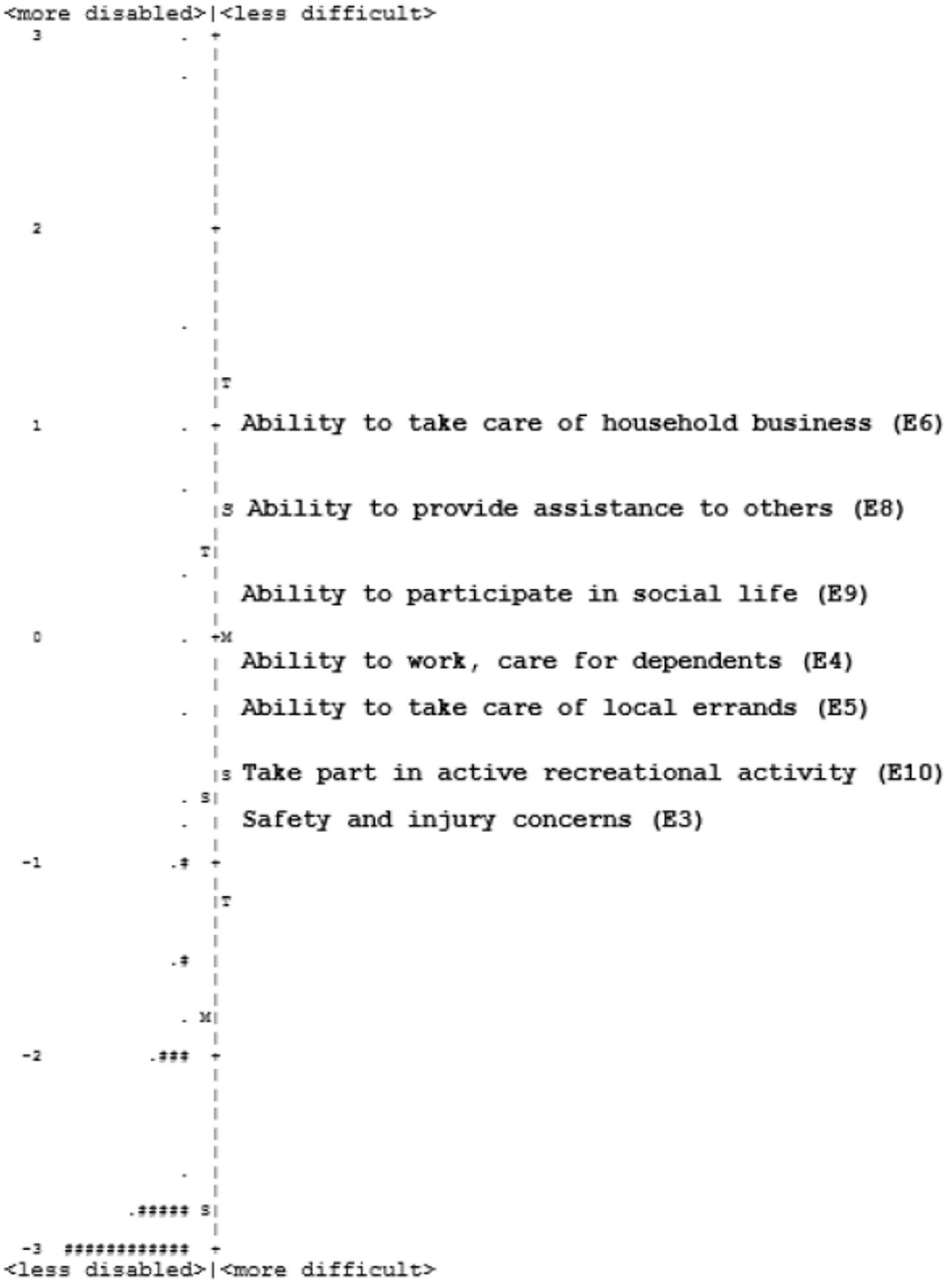
Person-item map for eCAPS. Respondents are shown on the left side of the dashed line and items are shown on the right side. Respondents who are less disabled (have higher visual function) and items that are more difficult (respondents are more likely to rate having greater difficulties with tasks) are located at the bottom. Each ‘#’ represents 12 respondents and each ‘.’ represents 1–11 respondents. M = mean, S = 1 standard deviation, T = 2 standard deviations. The scale is in logits.

The mean person location for eCAPS was -2.81, which is substantially lower than the mean item location set automatically at 0. This indicates poor targeting, meaning the tasks were relatively easy for the respondent ability. Fig 4 shows the distribution of person ability and item difficulty.

#### 3.3.6. Differential item functioning (DIF)

DIF occurred as a function of age with respect to item E8—‘ability to provide assistance to others’ (DIF contrast = 0.88, p = 0.0460, reported as more difficult for those 65-years-old or under). DIF did not occur as a function of gender.

## 4. Discussion

### Catquest-9SF

To pass a Rasch validation, a questionnaire must meet criteria with regards to several parameters: response threshold order, item fit, unidimensionality, targeting, precision, and differential item functioning. In our study, the Catquest-9SF fulfilled criteria for valid measurement. Catquest-9SF had ordered response thresholds and all items fit the Rasch model. Unidimensionality was confirmed with principal component analysis. The mean for patients was lower than the mean for items, meaning that tasks were easy relative to respondent ability. This is called mistargeting. However, there was adequate precision so the questionnaire can discriminate respondents effectively. There was no notable differential item functioning with respect to age, gender, or pre-operative visual acuity. This study confirms that the Catquest-9SF is suitable for assessing the visual function of patients with cataract in Peel region, Ontario, Canada. Use of this questionnaire in daily clinical practice will help identify patients who are more or less likely to benefit from cataract surgery and determine the optimal time for intervention based on the patient’s experiences with his/her vision.

The results of this study are similar to results of previous Catquest-9SF validation studies in other populations. Previous reports showed that Catquest-9SF had ordered response thresholds, unidimensionality confirmed by principal components analysis, and acceptable precision based on person separation index and person reliability [30]. Infit/outfit range was within 0.50–1.50 for these studies (except for two studies which had up to two misfitting items) [22, 26]. Furthermore, the mistargeting in this study (-1.43) is in line with previous validation reports that showed that Catquest-9SF was relatively easy pre-operatively [21, 23, 27, 41]. Like in other studies, the easiest question was C2 (‘recognizing faces’), meaning that respondents were more likely to report little or no difficulties with the task, and the most difficult question was Cb (‘satisfaction with vision’), meaning respondents were likely to report being dissatisfied [18, 19, 23, 24, 26, 28]. These similarities in findings in different countries and clinical settings indicates that the Catquest-9SF may be generalizable to other new populations as well.

Although Catquest-9SF is relatively short compared to other questionnaires such as the VF-14 questionnaire and the ADVS, it has been suggested that an even shorter questionnaire would be preferred to improve efficiency and practicality [11]. Usually this is achieved by removing redundant items. In this study, there was clustering of items C4 (‘walk on uneven ground’), C5 (‘do needlework/handicraft’), and C7 (‘carry out a hobby’), as well as items Ca (‘difficulties in daily life’), C3 (‘see prices when shopping’) and C6 (‘read text on television’), suggesting that some of these questions may be testing the same level of visual function (Fig 2). Similar patterns were reported in other validation studies, for example with item C4 and item C7 clustering around the same location on person-item maps [18, 20, 22, 27, 29]. Similarly, items Ca, C3, and C6 clustered in previous studies [19, 20]. The present study presents several combinations of items to shorten the Catquest 9SF. All combinations had ordered thresholds, were unidimensional, and free of significant DIF, however, they all had mistargeting indicating that the questions were too easy relative to respondent ability, like the 9-item version.

The main limitation of the shortened versions was inadequate precision. Three 8-item versions had acceptable precision: those that removed items C4, C5, or C7. For all other versions, the precision was inadequate to separate the sample into three strata (low, medium, and high visual function), however the minimum precision to divide the sample into two strata (low and high visual function) were met [34, 39]. Since low precision can be sample-specific (for example, caused by respondents having a narrow range of visual function or mistargeting), future validation studies with larger samples or in other populations in Canada may achieve the preferred precision for a 5- or 7-item questionnaire. The 5-item combination with the best precision was Cb, C1, C2, C3, C7 (Ca, C4, C5, C6 removed), with person separation 1.77 and person reliability 0.76. The 7-item combination with the best precision was Cb, C1, C2, C3, C5, C6, C7 (Ca and C4 removed), with person separation index 1.97 and person reliability 0.80. The 8-item combination with the best precision was Ca, Cb, C1, C2, C3, C5, C6, C7 (C4 removed), with person separation index 2.09 and person reliability 0.81. Therefore, based on these data, if we were to remove one question, it would be C4; two questions: Ca and C4; and four questions: Ca C4, C5, and C6. Catquest-9SF was shortened in two previous validation studies because of misfit (infit/outfit was greater than 1.5): Khadka et al. (China) removed item C5 (‘do needlework and handicraft’) and Nielsen et al. (Denmark) removed item C2 (‘recognize faces’) and item C4 (‘walk on uneven ground’) [22, 26]. Other validation studies did not report analysis of subsets of Catquest-9SF questions.

### eCAPS

eCAPS had 3 items that misfit the Rasch model (item calibration did not fit within the acceptable range of 0.50–1.50) and were excluded from the remainder of the analysis. Two of these items are expressed on a different rating scale with respect to the other items, which may explain the misfit. The 7-item eCAPS did not demonstrate adequate precision. Most subjects responded with ‘none/not threatened’ for almost all questions, indicating that the instrument is unable to separate respondents in this patient population. Although this lack of separation of respondents could be due to the tasks all being equally easy for the respondents, it could have also occurred because some tasks were not relevant to respondents’ daily lives. Since eCAPS does not have a ‘cannot decide’ option like Catquest-9SF, those who felt they did not participate in a certain activity chose ‘none/not threatened’ for that item. For example, a person with low visual function who did not have dependents chose the same answer for item E4 (‘ability to take care of dependents’) as someone who had high visual function and felt that taking care of his/her dependents was easy. This would provide an inaccurate score. The lack of precision limits the questionnaire’s usefulness in a clinical setting at the level of cataracts seen in this study population. Future considerations may include investigating which eCAPS items can be modified to become more difficult and more applicable to respondents, and/or adding a ‘cannot decide’ option. As shown by our sensitivity analysis for Catquest-9SF where we excluded the respondents who answered ‘cannot decide’ for any of the items, ‘missing data’ is handled by the Rasch model when such a response option is present.

### Conclusion

The Catquest-9SF demonstrated good psychometric properties and is suitable for assessing the visual function of patients with cataract in Peel Region, Canada. An 8-item shortened version may be considered as well, and even shorter versions may work, though they may have less precision. There was some mistargeting suggesting that the specified tasks were relatively easy to perform, which is in line with previous research. On the contrary, the eCAPS questionnaire is not sensitive in differentiating patients who had impaired vision. Future steps may include modification of the questionnaire to increase the difficulty of items, improve the reliability of the tasks to respondents, and/or changing the rating scale options.

A limitation of the study is that we only recruited patients within Peel Region, Ontario, Canada. A larger, multi-center sample may be useful in future studies. Furthermore, since we only had pre-operative questionnaires, future studies may also include post-operative questionnaire data in the Rasch analysis to assess responsiveness of the tool to cataract surgery.

## Supporting information

S1 Fig Catquest-9SF questionnaire. (DOCX)

S2 Fig eCAPS questionnaire (quality of life component). (DOCX)

S1 Table Description of psychometric properties assessed through Rasch analysis. (DOCX)

S2 Table Psychometric properties of combinations of 5 items (4 items removed—Two from each cluster), 7 items (2 items removed—One from each cluster), and 8 items (1 item removed—From either the first or second cluster). The best combination in each category is bolded. In the category with combinations of 8 items, the three combinations with acceptable precision were bolded.
(DOCX)

S1 Dataset (CSV)
