# Supplementary material for: Catquest-9SF questionnaire and eCAPS: Validation in a Canadian population
Source: PLoS One. 2020 Sep 25;15(9):e0237788. doi: 10.1371/journal.pone.0237788 (PMC7518613; doi:10.1371/journal.pone.0237788)
Supplement: S1 Fig — (DOCX) [file pone.0237788.s001.docx]

Catquest-9SF

| 1. Do you find that your sight at present in some way causes you difficulty in your everyday life? | | | | | | | | | |
| --- | --- | --- | --- | --- | --- | --- | --- | --- | --- |
| Yes,  very great difficulty | Yes,  great difficulty | | Yes, some difficulty | | No,  no difficulty | | | Cannot decide | |
| 1. Are you satisfied or dissatisfied with your present vision? | | | | | | | | | |
| Very dissatisfied | Fairly dissatisfied | | Fairly satisfied | | Very satisfied | | | Cannot decide | |
| 1. Do you have difficulty with the following activities because of your vision? If so, to what extent? In each row, mark only one cross, in the box which you think best corresponds to your situation. | | | | | | | | | |
|  | | Yes,  very great difficulty | | Yes,  great difficulty | | Yes, some difficulties | No,  no difficulty | | Cannot decide |
| Reading text in newspapers | |  | |  | |  |  | |  |
| Recognize the faces of people  you meet | |  | |  | |  |  | |  |
| Seeing prices the process of goods when shopping | |  | |  | |  |  | |  |
| Seeing to walk on uneven surfaces, e.g. cobblestones | |  | |  | |  |  | |  |
| Seeing to do handwork, woodworking, etc. | |  | |  | |  |  | |  |
| Reading subtitles on TV | |  | |  | |  |  | |  |
| Seeing to engage in an activity /hobby that you are interested in | |  | |  | |  |  | |  |
