# Supplementary material for: Catquest-9SF questionnaire and eCAPS: Validation in a Canadian population
Source: PLoS One. 2020 Sep 25;15(9):e0237788. doi: 10.1371/journal.pone.0237788 (PMC7518613; doi:10.1371/journal.pone.0237788)
Supplement: S2 Fig — (DOCX) [file pone.0237788.s002.docx]

eCAPS

*Please circle the level that most accurately describes the patient’s current situation.*

| **Extent of impairment in visual function**  (i.e., reading, watching TV, facial recognition, reading traffic signs, driving, seeing steps or curbs) | None | Mild/  moderate | Severe |
| --- | --- | --- | --- |
| **Other substantial disabilities**  (i.e. hearing loss, wheel chair use, partially reversible dementia) | None | Mild/  moderate | Severe |
| **Safety and injury concerns**  (i.e., falling, cooking, other risks related to impaired eyesight) | Not threatened | Mildly/ moderately threatened | Severely threatened |
| **Ability to work, care for dependents or work independently** | Not applicable/ not threatened | Mildly/ moderately threatened | Severely threatened |
| **Ability to take care of local errands**  (i.e., grocery shopping, going to the bank, library or dry cleaner) | Not threatened | Mildly/ moderately threatened | Severely threatened |
| **Ability to take care of household business and finances**  (i.e., managing money, paying bills) | Not threatened | Mildly/ moderately threatened | Severely threatened |
| **Ability to take care of own health**  (i.e., managing daily medications, scheduling and going to medical appointments) | Not threatened | Mildly/ moderately threatened | Severely threatened |
| **Ability to provide assistance to others**  (i.e., provide personal care, transportation and running errands for family members or friends) | Not threatened | Mildly/ moderately threatened | Severely threatened |
| **Ability to participate in social life and personal relationships**  (i.e., inviting people into your home or going out with others) | Not threatened | Mildly/ moderately threatened | Severely threatened |
| **Ability to take part in active recreational activity**  (i.e., jogging, hiking, swimming, golf, tennis, bowling) | Not threatened | Mildly/ moderately threatened | Severely threatened |

Lim et al. [unpublished]
