# Supplementary material for: Catquest-9SF questionnaire and eCAPS: Validation in a Canadian population
Source: PLoS One. 2020 Sep 25;15(9):e0237788. doi: 10.1371/journal.pone.0237788 (PMC7518613; doi:10.1371/journal.pone.0237788)
Supplement: S1 Table — (DOCX) [file pone.0237788.s003.docx]

| **Measure** | **Description** | **Acceptable Values** | **Conclusion if Acceptable Values** | **Causes of Unacceptable Values** |
| --- | --- | --- | --- | --- |
| **Category Threshold Order** | A category probability curve shows whether the category thresholds (three for Catquest-9SF, two for eCAPS) between the response categories are ordered. | Category thresholds are ordered. That is, the intersection between category probability curves 1 and 2 comes before the intersection of 2 and 3 (followed by the intersection of 3 and 4 for Catquest-9SF). | As expected, a respondent with lower visual function would consistently choose from the higher categories representing greater difficulties (and the opposite for those with higher visual function). | Disorder may occur when categories are difficult to differentiate, or when some categories are underused. |
| **Item Fit** | Infit and outfit mean squares (MNSQ) indicate how well the data match the Rasch model.  Outfit, or outlier-sensitive fit, assesses responses to items far from a person’s measures (e.g. person with high ability responding to easy item).  Infit, or inlier-sensitive or information-weighted fit, looks at responses of items closer to person’s measures (e.g. a person with medium ability responding to medium difficulty item) [41]. | A range of 0.50 to 1.50 is acceptable for both infit and outfit, with 1 being ideal [43]. | All items fit the model. There are no items with unexpected variance (underfit: values greater than 1.0) or overprediction (overfit: values less than 1.0), which causes inflated reliability results.  A scale with perfectly fitting items is more likely to be measuring only one trait – visual function [41]. | High outfit value can be due to a person with high visual function carelessly responding they have difficulty with an easy item, or vice versa [41].  Low outfit value can be due to imputed responses [41].  High infit value can be due to a person with medium visual function responding they have very great or no difficulties with a medium-difficulty item.  Low infit value can be due to a cumulative (or Guttman) pattern, which suggests that the fit is too good to be true and items are not independent [36]. |
| **Uni-dimensionality** | Principal Component Analysis (PCA) of the residuals is a definitive test for unidimensionality. That is, it tests whether the items on the questionnaire measure only one trait (in this case, visual function). | The raw variance explained by the measures should be >50% and/or comparable for the empirical calculation and by the model.  The unexplained variance explained by the first contrast should be <2.0 eigenvalue units, which is random noise level [40,42]. | All information in the data can be explained by visual function alone, and the unexplained part of the data (the residuals) is random noise [40]. | The unexplained part of the data is not random noise; the questionnaire items share the same patterns of unexpectedness, meaning that these items share a secondary trait - not just visual function. |
| **Precision** | Person Separation Index (PSI) indicates the number of statistically different levels of ability that can be distinguished by the questionnaire items.  Person Reliability (PR) is a measure of internal consistency or replicability of person ability across other items measuring the same latent trait [36]. | To discriminate between at least three levels of abilities (low, medium, and high), acceptable values are PSI >=2.0 and PR >=0.80 [34].  For two levels of abilities (low and high), PSI >= 1.5 and PR >=0.7 are acceptable [39]. | Acceptable PSI means the instrument can distinguish respondents who have different levels of visual function.  Acceptable PR means that persons estimated with high ability likely do have better visual function than those estimated with low ability [34]. | Low PSI and PR can be caused by respondents having a narrow range of visual function. It can also be due to not enough questionnaire items, not enough response options, and mistargeting [34]. |
| **Targeting** | A measure of how well the item difficulties match the person abilities. | A difference <1.0 between the item and person mean values indicates good targeting, with 0 being ideal [22,23,27,29]. | The tasks in the questionnaires are at an appropriate difficulty level for respondents. | A negative value means that the items are too easy (respondents are more likely to respond that they have no difficulties with the tasks), while a positive value means that the items are too difficult (respondents reported too much difficulty with the tasks). |
| **Differential Item Functioning (DIF)** | DIF occurs if respondents from different subgroups tend to respond differently to an item despite having the same true ability.  We assessed DIF for each item by age (<=65 or >65 years), gender (male or female), and/or pre-operative best-corrected visual acuity in the eye for surgery (grouped into vision under 20/50 or 20/50 and better). | DIF magnitude of <0.5 logits is considered small or absent, 0.50 to 1.0 logits is minimal, while >1.0 logits is notable [18].  A p-value of <0.05 is required for statistical significance. In this review, Rasch-Welch probabilities were used. | The items in the questionnaire are not biased across subgroups. | DIF is benign if it is due to intrinsic differences between the subgroups (e.g. one subgroup does a task more frequently, which affects responses).  DIF can be adverse if it is caused by item bias such as poor wording [37,38]. |
