# Supplementary material for: Catquest-9SF questionnaire and eCAPS: Validation in a Canadian population
Source: PLoS One. 2020 Sep 25;15(9):e0237788. doi: 10.1371/journal.pone.0237788 (PMC7518613; doi:10.1371/journal.pone.0237788)
Supplement: S2 Table — The best combination in each category is bolded. In the category with combinations of 8 items, the three combinations with acceptable precision were bolded. (DOCX) [file pone.0237788.s004.docx]

| Combinations of 5 items | | | | | | | | | | | | |
| --- | --- | --- | --- | --- | --- | --- | --- | --- | --- | --- | --- | --- |
| Removed Items | **Ca, C4, C5, C6** | Ca, C3 , C5, C7 | Ca, C3, C4, C7 | Ca, C5, C6, C7 | Ca, C3, C4, C5 | | Ca, C4, C6, C7 | C3, C4, C5, C6 | | C3, C5, C6, C7 | C3, C3, C6, C7 |  |
| Infit Range | **0.86-1.35** | 0.84-1.24 | 0.80-1.23 | 0.85-1.37 | 0.83-1.24 | | 0.82-1.37 | 0.76-1.40 | | 0.72-1.36 | 0.75-1.44 |  |
| Outfit Range | **0.80-1.23** | 0.79-1.15 | 0.76-1.29 | 0.80-1.28 | 0.85-1.05 | | 0.77-1.35 | 0.82-1.19 | | 0.77-1.23 | 0.82-1.17 |  |
| Variance explained by the measures for empirical calculation; for model (%) | **66.8 ; 67.0** | 64.8 ; 65.0 | 64.3 ; 64.6 | 65.0 ; 64.8 | 65.3 ; 65.8 | | 64.7 ; 64.8 | 66.1 ; 66.4 | | 64.1 ; 64.1 | 63.3 ; 63.7 |  |
| Unexplained variance explained by the first contrast (eigenvalue units) | **1.61** | 1.42 | 1.44 | 1.72 | 1.44 | | 1.61 | 1.44 | | 1.33 | 1.49 |  |
| Person Separation Index (PSI) | **1.77** | 1.62 | 1.67 | 1.64 | 1.71 | | 1.67 | 1.68 | | 1.58 | 1.58 |  |
| Person Reliability (PR) | **0.76** | 0.72 | 0.74 | 0.73 | 0.75 | | 0.74 | 0.74 | | 0.71 | 0.71 |  |
| Difference between mean for persons and mean for items | **-1.37** | -1.37 | -1.35 | -1.30 | -1.40 | | -1.28 | -1.50 | | -1.44 | -1.39 |  |
| Combinations of 7 items | | | | | | | | | | | | |
| Removed Items | Ca, C7 | Ca, C5 | **Ca, C4** | C3, C7 | | C3, C5 | C3, C4 | C6, C7 | | C6, C5 | C6, C4 |  |
| Infit Range | 0.81-1.27 | 0.88-1.34 | **0.90-1.31** | 0.78-1.29 | | 0.74-1.33 | 0.81-1.31 | 0.72-1.36 | | 0.70-1.44 | 0.78-1.38 |  |
| Outfit Range | 0.80-1.25 | 0.82-1.31 | **0.82-1.37** | 0.80-1.20 | | 0.82-1.28 | 0.83-1.06 | 0.81-1.28 | | 0.80-1.40 | 0.82-1.15 |  |
| Variance explained by the measures for empirical calculation; for model (%) | 60.8 ; 61.2 | 61.1 ; 61.7 | **61.6 ; 62.4** | 59.6 ; 60.2 | | 60.2 ; 60.9 | 60.9; 61.7 | 59.6 ; 59.9 | | 61.3 ; 61.7 | 61.9 ; 62.6 |  |
| Unexplained variance explained by the first contrast (eigenvalue units) | 1.65 | 1.66 | **1.63** | 1.64 | | 1.54 | 1.70 | 1.66 | | 1.65 | 1.67 |  |
| Person Separation Index (PSI) | 1.87 | 1.90 | **1.97** | 1.82 | | 1.86 | 1.91 | 1.83 | | 1.88 | 1.94 |  |
| Person Reliability (PR) | 0.78 | 0.78 | **0.80** | 0.77 | | 0.77 | 0.79 | 0.77 | | 0.78 | 0.79 |  |
| Difference between mean for persons and mean for items | -1.34 | -1.38 | **-1.39** | -1.43 | | -1.47 | -1.48 | -1.37 | | -1.43 | -1.44 |  |
| Combinations of 8 items | | | | | | | | |  |  |  |  |
| Removed Item | Ca | C3 | **C4** | **C5** | C6 | | **C7** |  |  |  |  |  |
| Infit Range | 0.85-1.30 | 0.78-1.29 | **0.79-1.35** | **0.72-1.40** | 0.73-1.36 | | **0.75-1.33** |  |  |  |  |  |
| Outfit Range | 0.80-1.32 | 0.80-1.27 | **0.86-1.18** | **0.83-1.38** | 0.82-1.34 | | **0.84-1.31** |  |  |  |  |  |
| Variance explained by the measures for empirical calculation; for model (%) | 59.6 ; 60.4 | 58.5 ; 59.4 | **60.4 ; 61.1** | **59.7 ; 60.3** | 59.2 ; 59.9 | | **59.2 ; 59.5** |  |  |  |  |  |
| Unexplained variance explained by the first contrast (eigenvalue units) | 1.63 | 1.69 | **1.67** | **1.65** | 1.69 | | **1.69** |  |  |  |  |  |
| Person Separation Index (PSI) | 1.97 | 1.93 | **2.09** | **2.03** | 1.94 | | **2.00** |  |  |  |  |  |
| Person Reliability (PR) | 0.79 | 0.79 | **0.81** | **0.81** | 0.79 | | **0.80** |  |  |  |  |  |
| Difference between mean for persons and mean for items | -1.43 | -1.51 | **-1.40** | **-1.39** | -1.46 | | **-1.36** |  |  |  |  |  |
